# Gender‐based violence during pregnancy and adverse perinatal outcomes in an urban low‐ and middle‐income setting: A prospective cohort study

**DOI:** 10.1002/ijgo.71189

**Published:** 2026-06-24

**Authors:** Elsa Romelia Moreno‐Verduzco, Javier Perez Duran, Irma Eloisa Monroy‐Muñoz, Juan Mario Solis‐Paredes, Salvador Espino‐y‐Sosa, Raigam Martinez‐Portilla, Johnatan Torres‐Torres

**Affiliations:** ^1^ Department of Reproductive and Perinatal Health Research Instituto Nacional de Perinatologia Isidro Espinosa de los Reyes Mexico City Mexico; ^2^ Department of Bioinformatics Instituto Nacional de Perinatologia Isidro Espinosa de los Reyes Mexico City Mexico; ^3^ Iberoamerican Research Network in Obstetrics, Gynecology and Translational Medicine Mexico City Mexico

**Keywords:** absolute risk, adverse perinatal outcomes, domestic violence, gender‐based violence, low‐ and middle‐income countries, pregnancy, stillbirth

## Abstract

**Objective:**

To evaluate the association between gender‐based violence (GBV) assessed in early pregnancy and adverse perinatal outcomes using relative and absolute effect measures in an urban low‐ and middle‐income setting.

**Methods:**

We conducted a prospective cohort study including 3890 singleton pregnancies recruited from two public healthcare institutions in Mexico City between October 2020 and April 2023. GBV was assessed at first‐trimester enrollment using a standardized screening procedure and analyzed as any reported violence versus none. The primary outcome was a composite of low birth weight, fetal growth restriction, or perinatal mortality. Associations were estimated using Poisson regression with robust variance to obtain adjusted risk ratios and risk differences. Secondary analyses examined fetal growth restriction, stillbirth, and neonatal mortality.

**Results:**

GBV was reported by 162 (4.16%) participants. After adjustment for sociodemographic factors, GBV was associated with higher risk of the composite adverse perinatal outcome (adjusted risk ratio [aRR] 1.23, 95% [confidence interval] CI: 1.06–1.44). The adjusted absolute risk difference was +0.099 (95% CI: 0.020–0.177), corresponding to approximately 99 additional adverse outcomes per 1000 births among women reporting GBV. GBV was not significantly associated with isolated fetal growth restriction but was associated with increased odds of stillbirth; no statistically significant association was observed with neonatal mortality.

**Conclusion:**

In this urban cohort, GBV reported at first‐trimester screening was associated with a clinically meaningful increase in adverse perinatal outcomes, largely driven by stillbirth.

## 1 INTRODUCTION

Gender‐based violence (GBV) remains a pervasive global public health and human rights problem.^1^ According to recent estimates from the WHO and the United Nations, nearly 840 million women—approximately one in three worldwide—have experienced physical, sexual, and/or psychological violence by an intimate partner or non‐partner during their lifetime, underscoring the extensive global burden of GBV.^2^ Although GBV occurs across all socioeconomic contexts, its burden is disproportionately concentrated in low‐ and middle‐income countries (LMICs), where structural inequality, limited access to protective services, and constrained health systems intersect to exacerbate women's vulnerability.^3^ Pregnancy represents a particularly sensitive period, during which exposure to violence may have profound consequences not only for maternal wellbeing, but also for fetal development and perinatal survival.

Growing evidence, including recent systematic reviews and meta‐analyses, suggests that intimate partner violence or gender‐based violence during pregnancy is associated with adverse birth outcomes, including low birth weight, preterm birth, and stillbirth.^4^, ^5^ These adverse outcomes may cluster and co‐occur during the perinatal period, yet they represent clinically and biologically distinct events. Proposed mechanisms include chronic psychosocial stress, neuroendocrine and inflammatory dysregulation, nutritional compromise, reduced engagement with prenatal care, and increased exposure to harmful health behaviors.^6^

Importantly, much of the existing literature on GBV and perinatal health, including recent meta‐analyses, has relied primarily on relative measures of association, particularly odds ratios or adjusted odds ratios, without translating these findings into absolute risks or risk differences.^4^, ^5^, ^7^, ^8^, ^9^ While relative estimates are essential for etiological inference, they provide limited information for clinicians and policymakers tasked with prioritizing interventions and allocating scarce resources.^7^, ^8^, ^9^ As a result, the population‐level impact of GBV on perinatal outcomes—and its potential contribution to preventable perinatal morbidity and mortality—remains underappreciated. In addition, under‐ascertainment of GBV due to stigma and safety concerns is common and may lead to systematic underestimation of its true impact.

Furthermore, previous studies have often examined single outcomes in isolation or have not adequately accounted for the complex interplay between GBV and broader social determinants of health, such as education, poverty, and social support. This has limited the ability to distinguish whether GBV functions as an independent determinant of perinatal risk or merely as a marker of underlying socioeconomic disadvantage. Clarifying this distinction is particularly relevant in urban LMIC contexts, where traditional socioeconomic gradients may not fully capture women's lived experiences of vulnerability.^9^, ^10^, ^11^, ^12^, ^13^

To address these gaps, we analyzed data from a large, prospective maternal cohort in Mexico City to evaluate the association between GBV reported at first‐trimester screening, reflecting recent exposure around the periconceptional and early gestational period, and adverse perinatal outcomes using both relative and absolute effect measures, while accounting for key sociodemographic and social determinants. The objectives of this study were: (1) to characterize the prevalence and social correlates of reported gender‐based violence assessed in early pregnancy; (2) to assess the association between GBV and a composite adverse perinatal outcome encompassing low birth weight, fetal growth restriction, or perinatal mortality; and (3) to quantify the absolute impact of GBV on perinatal risk in an urban LMIC setting.

## 2 MATERIALS AND METHODS

### 2.1 Study design and population

This was a prospective cohort analysis of singleton pregnancies enrolled in a maternal cohort in Mexico City. Pregnant women were consecutively recruited at 11–13.6 weeks of gestation from two public healthcare institutions—the Romero Rubio Primary Care Health Center and the Instituto Nacional de Perinatología “Isidro Espinosa de los Reyes,” a national tertiary referral center—between October 2020 and April 2023. Exposure to gender‐based violence was assessed at first‐trimester enrollment, before ascertainment of the perinatal outcomes analyzed in this study. Participants were followed prospectively through pregnancy, delivery, and the postpartum/neonatal period using standardized cohort procedures and medical record review.

Women were eligible for inclusion if they were aged ≥18 years and had a confirmed singleton pregnancy. Exclusion criteria included major fetal structural or chromosomal abnormalities, withdrawal of informed consent, or loss to follow‐up before delivery. The analytic cohort included participants with available GBV assessment and perinatal outcome data.

### 2.2 Ethical approval and informed consent

The study protocol was reviewed and approved by the Institutional Review Board and Ethics Committee of the Instituto Nacional de Perinatología “Isidro Espinosa de los Reyes” (approval no. 2021‐1‐38). All procedures were conducted in accordance with the Declaration of Helsinki and its later amendments. Participants were informed that they were being invited to participate in a prospective research cohort, including standardized data collection and follow‐up during pregnancy and the postpartum period. Written informed consent was obtained from all participants prior to enrollment and before any study‐specific procedures, including the gender‐based violence assessment. Participation was voluntary, and refusal to participate or to answer specific questions did not affect clinical care.

### 2.3 Assessment of gender‐based violence

Exposure to GBV was assessed during first‐trimester enrollment (11–13.6 weeks of gestation) using a standardized family violence screening procedure implemented as part of the research cohort protocol, rather than as routine clinical care at the first prenatal visit.^1^ The assessment was performed by trained research personnel in a private and confidential setting, following standardized safety procedures to facilitate disclosure and reduce reporting bias. In this cohort, GBV was prospectively recorded in the analytic database as a single binary screening result, defined as any reported violence versus none. Subtype‐specific information on emotional, physical, or sexual violence was not recorded separately; therefore, the exposure should be interpreted as a global indicator of reported GBV at early pregnancy research screening rather than as a measure of a specific violence subtype, severity, or chronicity. Accordingly, subtype‐specific relative risks could not be estimated.

Participants were asked to report any experience of violence occurring within the preceding 12 months. The questionnaire did not capture exact dates or frequency of individual violent events; therefore, the exposure cannot establish the precise timing of each event in relation to conception or pregnancy. The measure is best interpreted as recent GBV identified at first‐trimester screening, which may include violence occurring before conception, during the periconceptional period, and/or during early pregnancy. This screening point was selected because early gestation represents a critical window for placental development, implantation, and vascular remodeling, processes that may plausibly mediate downstream perinatal risk. For the purposes of the present analysis, GBV exposure was analyzed as a binary screening indicator within the cohort. The study was not designed to assess violence subtype, persistence, frequency, or severity across pregnancy.

To ensure participant safety and minimize underreporting, the questionnaire was administered only when the participant was alone, without partners or accompanying family members present. Interviews were conducted in a private clinical setting by trained female research staff with prior experience in sensitive data collection. Participants were informed that disclosure was voluntary and would not affect their clinical care. When ongoing violence or current safety concerns were disclosed, participants were provided with information on institutional and community‐based support resources according to local ethical guidelines.

### 2.4 Sociodemographic and social determinants

Sociodemographic variables included maternal age, marital status (married, single, cohabiting), and educational attainment. Educational level was initially recorded in six categories (illiterate, primary, secondary, high school, university, postgraduate) and was subsequently collapsed into three analytically meaningful categories (low: illiterate/primary/secondary; medium: high school; high: university/postgraduate) to avoid sparse data and model instability.

Poverty and extreme poverty were defined according to the methodology of the National Council for the Evaluation of Social Development Policy (CONEVAL), incorporating income and social deprivation indicators. Household overcrowding was assessed using the WHO definition of more than three persons per room. Social support was measured using the Medical Outcomes Study Social Support Survey and categorized into low, medium, and high support levels based on standardized cutoffs.

A social vulnerability index was assigned at the ecological level based on participants' residential postal codes, classifying areas into low, medium, or high vulnerability using national thresholds.

### 2.5 Clinical and obstetric variables

Clinical data were abstracted from medical records and included parity, mode of conception, pregestational body mass index, smoking and alcohol use during pregnancy, illicit drug use, and pre‐existing medical conditions (including diabetes, chronic hypertension, lupus, antiphospholipid syndrome, hypothyroidism, polycystic ovary syndrome, and maternal heart disease). Obstetric history included prior pre‐eclampsia and prior fetal growth restriction.

### 2.6 Outcomes

The primary outcome was a composite adverse perinatal outcome, defined as the occurrence of at least one of the following: low birth weight (<2500 g), fetal growth restriction, or perinatal mortality. Fetal growth restriction was defined antenatally as an estimated fetal weight below the third percentile, or below the 10th percentile in the presence of abnormal Doppler findings, according to institutional ultrasound protocols. Secondary outcomes included fetal growth restriction analyzed independently and perinatal mortality disaggregated into stillbirth and neonatal mortality. Stillbirth was defined as fetal death at ≥28 weeks of gestation, and neonatal mortality was defined as death within the first 28 days of life, in accordance with WHO definitions.

### 2.7 Statistical analysis

Baseline characteristics are summarized according to reported gender‐based violence status. Continuous variables were compared using two‐sample *t*‐tests or non‐parametric equivalents, as appropriate, and categorical variables were compared using Pearson's *χ* ^2^ test or Fisher exact test.

Multivariable logistic regression was used to identify social determinants independently associated with reported gender‐based violence, with results expressed as adjusted odds ratios (aORs) and 95% confidence intervals (CIs). Covariates included maternal age, marital status, education, poverty, extreme poverty, social support, and household overcrowding.

For the primary composite outcome, Poisson regression with robust variance and a log link was employed to estimate adjusted risk ratios (aRRs), given the relatively high outcome frequency. Adjusted absolute risks were obtained by marginal standardization of the fitted Poisson models using predictive margins, and adjusted risk differences were calculated as contrasts of predicted risks between exposure groups. CIs for risk differences were derived using the delta method.

For secondary outcomes, fetal growth restriction was analyzed using Poisson regression with robust variance. Stillbirth was analyzed using penalized logistic regression (Firth method) due to separation in conventional logistic models, whereas neonatal mortality was analyzed using conventional logistic regression, as models converged without evidence of separation or instability.

All multivariable models were adjusted a priori for maternal age, marital status, education, poverty, social support, and household overcrowding, based on clinical relevance and prior literature. Statistical significance was defined as a two‐sided *P* value less than 0.05.

Given the potential for underreporting of gender‐based violence, a non‐differential exposure misclassification sensitivity analysis was conducted to evaluate the impact of under‐ascertainment. Plausible scenarios were explored assuming sensitivity values ranging from 0.30 to 0.60 and specificity values between 0.97 and 0.99. Corrected adjusted effect estimates were derived analytically, and uncertainty for corrected risk ratio estimates was assessed using bootstrap resampling. All analyses were performed using Stata Statistical Software, version 18 (StataCorp, College Station, Texas, USA).

This study is reported in accordance with the Strengthening the Reporting of Observational Studies in Epidemiology (STROBE) guideline for cohort studies (Table S1).

## 3 RESULTS

### 3.1 Study population and prevalence of gender‐based violence

A total of 3890 pregnant women were included in the analytic cohort, of whom 162 (4.16%) reported exposure to gender‐based violence. Baseline sociodemographic, clinical, and obstetric characteristics stratified by reported violence status are summarized in Table 1.

**TABLE 1:** Baseline characteristics by reported gender‐based violence.

Characteristic	No violence (*n* = 3728)	Violence (*n* = 162)	*P* value
**Sociodemographic characteristics**			
Maternal age, years (mean ± SD)	31.04 ± 7.83	30.59 ± 7.93	0.481
Marital status, *n* (%)			0.854
Married	1115 (29.9)	50 (30.9)	
Single	971 (26.0)	39 (24.1)	
Cohabiting	1642 (44.0)	73 (45.1)	
Education, *n* (%)			0.013
Illiterate	70 (1.9)	0 (0.0)	
Primary	330 (8.9)	9 (5.6)	
Secondary	1296 (34.8)	77 (47.5)	
High school	1316 (35.3)	46 (28.4)	
University	650 (17.4)	28 (17.3)	
Postgraduate	66 (1.8)	2 (1.2)	
Poverty, *n* (%)	639 (17.1)	26 (16.0)	0.718
Extreme poverty, *n* (%)	166 (4.5)	10 (6.2)	0.302
Vulnerability index, *n* (%)			0.282
Low	1705 (45.7)	64 (39.5)	
Medium	1412 (37.9)	67 (41.4)	
High	611 (16.4)	31 (19.1)	
Social support, *n* (%)			0.898
High	1181 (31.7)	53 (32.7)	
Medium	1899 (50.9)	83 (51.2)	
Low	648 (17.4)	26 (16.0)	
Overcrowding, *n* (%)	159 (4.3)	9 (5.6)	0.429
**Clinical characteristics, behaviors, and obstetric history**			
Pre‐pregnancy BMI (mean ± SD)	26.77 ± 4.77	27.41 ± 4.30	0.095
Nulliparity, *n* (%)	1257 (33.7)	58 (35.8)	0.583
Pre‐existing diabetes, *n* (%)	197 (5.3)	3 (1.9)	0.053
Chronic hypertension, *n* (%)	109 (2.9)	6 (3.7)	0.566
Lupus, *n* (%)	26 (0.7)	1 (0.6)	0.904
APS, *n* (%)	19 (0.5)	0 (0.0)	0.362
Hypothyroidism, *n* (%)	371 (10.0)	14 (8.6)	0.585
Maternal heart disease, *n* (%)	11 (0.3)	1 (0.6)	0.469
Polycystic ovary syndrome, *n* (%)	82 (2.2)	2 (1.2)	0.408
Smoking during pregnancy, *n* (%)	264 (7.1)	15 (9.3)	0.293
Alcohol use during pregnancy, *n* (%)	65 (1.7)	6 (3.7)	0.068
Illicit drug use (cocaine/heroin), *n* (%)	69 (1.9)	1 (0.6)	0.248
In vitro fertilization (IVF), *n* (%)	40 (1.1)	1 (0.6)	0.578
Ovulation induction, *n* (%)	30 (0.8)	1 (0.6)	0.793
Prior pre‐eclampsia, *n* (%)	260 (7.0)	11 (6.8)	0.928
Prior fetal growth restriction, *n* (%)	230 (6.2)	8 (4.9)	0.522
Family history of pre‐eclampsia (mother), *n* (%)	181 (4.9)	10 (6.2)	0.447

*Note*: BMI, calculated as weight in kilograms divided by the square of height in meters. Values are *n* (%) unless otherwise indicated. Percentages are column percentages within violence strata. *P* values from Pearson *χ* ^2^ tests for categorical variables and two‐sample *t*‐tests for continuous variables; non‐parametric tests yielded similar results.

Abbreviations: APS, antiphospholipid syndrome; BMI, body mass index; IVF, in vitro fertilization; SD, standard deviation.

Marital status distributions were comparable between women with and without reported violence (*P* = 0.854). In unadjusted comparisons, educational attainment differed significantly by violence status (*P* = 0.013), with a higher proportion of women with secondary education and a lower proportion with high school education among those reporting violence. In contrast, no statistically significant differences were observed across groups for poverty (*P* = 0.718), extreme poverty (*P* = 0.302), social vulnerability index category (*P* = 0.282), social support (*P* = 0.898), or household overcrowding (*P* = 0.429).

Maternal age and pre‐pregnancy body mass index (BMI, calculated as weight in kilograms divided by the square of height in meters) were similar between groups. Women without reported violence had a mean maternal age of 31.04 years (standard deviation [SD] 7.83) compared with 30.59 years (SD 7.93) among those reporting violence (*P* = 0.481). Mean pre‐pregnancy BMI was 26.77 (SD 4.77) in women without violence and 27.41 (SD 4.30) in those with reported violence (*P* = 0.095).

Baseline clinical characteristics, health behaviors, and obstetric history were largely comparable by violence status. The prevalence of nulliparity, chronic hypertension, autoimmune disease, thyroid disorders, smoking, alcohol use, illicit drug use, assisted reproductive technologies, and prior obstetric complications—including pre‐eclampsia and fetal growth restriction—did not differ significantly between women with and without reported violence.

### 3.2 Social determinants associated with gender‐based violence

Adjusted associations between social determinants and reported gender‐based violence are presented in Table 2. In multivariable logistic regression analyses adjusting for maternal age, marital status, education, poverty, extreme poverty, social support, and household overcrowding, medium educational attainment (high school) was associated with lower odds of reporting gender‐based violence compared with low educational attainment (illiterate/primary/secondary) (aOR 0.69, 95% CI: 0.48–0.99, *P* = 0.045).

**TABLE 2:** Social determinants associated with reported gender‐based violence.

Predictor	Adjusted OR (95% CI)	*P* value
Maternal age (per 1 year)	0.99 (0.97–1.01)	0.469
Marital status (ref. married)		
Single	0.89 (0.58–1.37)	0.606
Cohabiting	1.00 (0.69–1.44)	0.982
Education (3‐level) (ref. Low: illiterate/primary/secondary)		
Medium (high school)	0.69 (0.48–0.99)	0.045
High (university/postgraduate)	0.82 (0.54–1.25)	0.360
Poverty (yes vs. no)	0.68 (0.38–1.21)	0.187
Extreme poverty (yes vs. no)	1.74 (0.78–3.92)	0.178
Overcrowding (yes vs. no)	1.29 (0.64–2.62)	0.474
Social support (ref. high)		
Low	0.89 (0.55–1.44)	0.634
Medium	0.97 (0.68–1.38)	0.877

*Note*: aORs derived from multivariable logistic regression. Models were adjusted for maternal age, marital status, education, poverty, extreme poverty, social support, and household overcrowding. Low education served as the reference category to avoid sparse data and separation.

Abbreviations: aORs, adjusted odds ratios; CI, confidence interval; OR, odds ratio.

In contrast, high educational attainment (university/postgraduate) was not significantly associated with reported violence when compared with low education (aOR 0.82, 95% CI: 0.54–1.25, *P* = 0.360). Maternal age, marital status, poverty, extreme poverty, household overcrowding, and levels of social support were not independently associated with reported gender‐based violence in the fully adjusted model.

### 3.3 Association between gender‐based violence and adverse perinatal outcomes

In adjusted Poisson regression with robust variance, reported gender‐based violence was associated with a higher risk of the composite adverse perinatal outcome, defined as low birth weight, fetal growth restriction, or perinatal mortality (aRR 1.23, 95% CI: 1.06–1.44, *P* = 0.007), after adjustment for maternal age, marital status, education, poverty, social support, and household overcrowding (Table 3 and Figure 1).

**TABLE 3:** Association between reported gender‐based violence and perinatal outcomes.

Outcome	Effect measure	Adjusted estimate (95% CI)	*P* value
Primary outcome			
Composite adverse perinatal outcome^a^	RR	1.23 (1.06–1.44)	0.007
Adjusted absolute risks (predictive margins)			
No reported violence	Risk	0.422 (0.407–0.438)	—
Reported violence	Risk	0.521 (0.444–0.598)	—
Adjusted risk difference	—	+0.099 (0.020–0.177)	0.014
Secondary outcomes			
Fetal growth restriction	RR	1.32 (0.73–2.37)	0.357
Stillbirth	OR	4.10 (1.30–12.93)	0.016
Neonatal mortality	OR	1.93 (0.69–5.36)	0.209

*Note*: aRRs were estimated using Poisson regression with robust variance; aORs were estimated using logistic regression. Stillbirth was analyzed using penalized logistic regression (Firth method) due to the low number of events and evidence of separation in standard models. Models were adjusted for maternal age, marital status, education, poverty, social support, and household overcrowding.

Abbreviations: aORs, adjusted odds ratios; aRRs, adjusted risk ratios; CI, confidence interval; OR, odds ratio; RR, risk ratio.

^a^ Composite adverse perinatal outcome defined as low birth weight, fetal growth restriction, or perinatal mortality.

**FIGURE 1:**
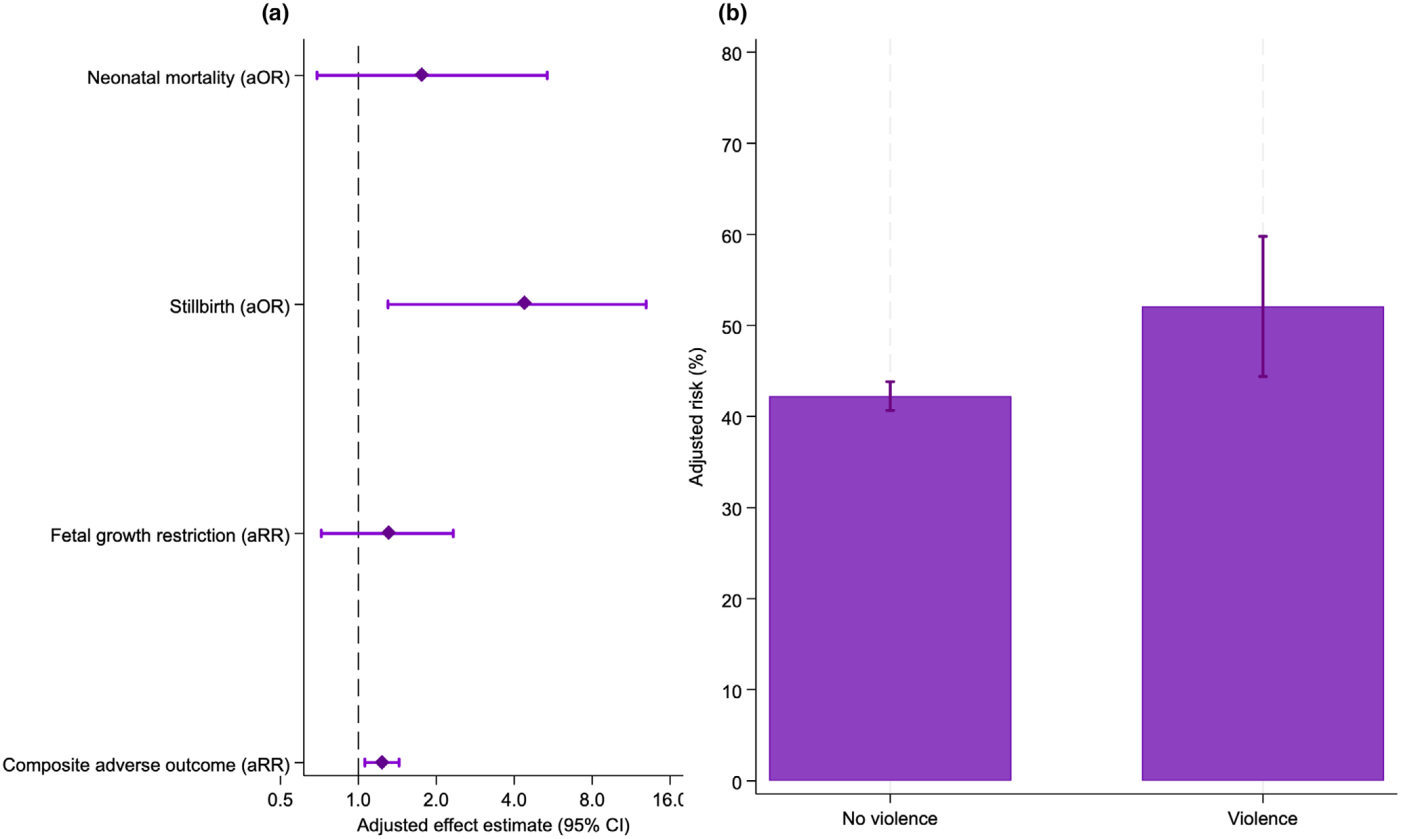
Association of gender‐based violence with perinatal outcomes. (a) Shows adjusted relative effect estimates (risk ratios [RRs] and odds ratios [ORs] with 95% confidence intervals [CIs]) for the composite adverse perinatal outcome, fetal growth restriction, stillbirth, and neonatal mortality. Stillbirth was modeled using penalized logistic regression (Firth) due to separation in conventional logistic models. (b) Displays adjusted absolute risks for the composite adverse perinatal outcome by reported violence status, obtained by marginal standardization of the fitted Poisson models. The adjusted risk difference was +9.9 percentage points (95% CI: 2.0–17.7), corresponding to approximately 99 additional adverse outcomes per 1000 births among women reporting gender‐based violence.

In terms of absolute effects, the adjusted risk of the composite adverse outcome was 0.422 (95% CI: 0.407–0.438) among women without reported violence and 0.521 (95% CI: 0.444–0.598) among those reporting violence, corresponding to an adjusted risk difference of +0.099 (95% CI: +0.020 to +0.177, *P* = 0.014). This translates into approximately 99 additional adverse outcomes per 1000 births among women reporting violence compared with those not reporting violence.

In secondary analyses, reported violence was not significantly associated with fetal growth restriction when examined as an isolated outcome in adjusted Poisson regression (aRR 1.32, 95% CI: 0.73–2.37, *P* = 0.357). Perinatal mortality was further disaggregated into stillbirth and neonatal mortality. In a penalized logistic regression model, reported gender‐based violence was associated with higher odds of stillbirth (aOR 4.10, 95% CI: 1.30–12.93). In contrast, no statistically significant association was observed between reported violence and neonatal mortality in adjusted logistic regression (aOR 1.93, 95% CI: 0.69–5.36). These analyses were considered exploratory given the limited number of events.

Across plausible non‐differential exposure misclassification scenarios, corrected adjusted RR estimates consistently remained greater than unity, ranging from 1.31 to 1.86. Under assumptions of high specificity (Sp = 0.99), which are plausible for a structured violence questionnaire, corrected RR estimates were stable at approximately 1.31–1.34 across sensitivity values from 0.30–0.60, indicating that the observed association between reported gender‐based violence and adverse perinatal outcomes is robust to under‐ascertainment of exposure (Table S2).

## 4 DISCUSSION

### 4.1 Main findings

In this large prospective urban cohort from Mexico City, reported exposure to gender‐based violence assessed in early pregnancy was associated with an increased risk of adverse perinatal outcomes. After adjustment for key sociodemographic determinants, women reporting gender‐based violence experienced a 23% higher risk of a composite adverse perinatal outcome including low birth weight, fetal growth restriction, or perinatal mortality. Importantly, this relative association translated into a substantial absolute difference, corresponding to approximately 100 additional adverse outcomes per 1000 births among women reporting violence. These findings suggest that gender‐based violence may represent an important and potentially actionable determinant of perinatal health in urban low‐ and middle‐income settings.

Disaggregation of the composite outcome provided additional clinical insight. When evaluated as a standalone endpoint, fetal growth restriction did not account for the observed association; instead, the association was driven by more severe outcomes. In secondary analyses, reported gender‐based violence was associated with increased odds of stillbirth, whereas no statistically significant association was observed with neonatal mortality. Although based on a limited number of events and therefore exploratory in nature, this pattern pointed toward an excess risk of stillbirth rather than neonatal mortality, defined in this study as death within the first 28 days of life. This finding raises the possibility that violence exposure during pregnancy may preferentially affect intrauterine pathways critical for fetal survival, even in settings with access to prenatal and neonatal care. Importantly, the absence of a statistically significant association with neonatal mortality should not be interpreted as evidence that postnatal risks are absent. The present study did not assess morbidity or mortality beyond the neonatal period, nor did it evaluate early childhood health, developmental, injury‐related, or safeguarding outcomes.

The timing of violence exposure warrants careful consideration. In this study, gender‐based violence was assessed during the first trimester, capturing recent experiences that may have occurred before conception, during the periconceptional period, and/or during early pregnancy. Although violence occurring several months prior to conception may appear temporally distant from pregnancy outcomes, accumulating evidence indicates that psychosocial stress and trauma during the periconceptional and early gestational period can exert lasting effects on placental development, vascular adaptation, and fetal programming.^6^, ^14^, ^15^ These early biological processes are established before clinical manifestations of pregnancy complications and may influence downstream risks even in the absence of continued exposure later in gestation. Importantly, our study does not assume persistence of violence throughout pregnancy; rather, it highlights early exposure as a clinically and biologically relevant window.

### 4.2 Comparison with existing literature

The observed association between gender‐based violence and adverse perinatal outcomes is consistent with prior literature linking chronic psychosocial stress, unsafe domestic environments, and structural vulnerability to impaired maternal–fetal health. Proposed mechanisms include sustained activation of stress‐response pathways, placental dysfunction, inflammatory and vascular dysregulation, and reduced maternal engagement with prenatal care.^4^, ^5^, ^16^ However, previous studies have frequently analyzed perinatal outcomes in isolation or have relied primarily on relative effect measures without reporting absolute risks, limiting their translational relevance.

By integrating both relative and absolute effect estimates, our findings extend existing evidence by demonstrating that the impact of violence exposure is not only statistically significant but also clinically meaningful at the population level. The absence of a significant association with isolated fetal growth restriction aligns with heterogeneous findings reported in prior studies and suggests that violence may exert cumulative or non‐specific effects that manifest preferentially as severe outcomes such as stillbirth rather than as isolated growth impairment.^5^, ^17^, ^18^

### 4.3 Clinical and policy implications

From a clinical perspective, these findings underscore the relevance of gender‐based violence as a factor that may contribute to severe perinatal outcomes, particularly stillbirth. While causality cannot be inferred from observational data, the magnitude of the observed absolute risk difference highlights the potential importance of identifying women exposed to violence during pregnancy. Although GBV screening was implemented in this study as part of a research cohort protocol rather than routine clinical care, our findings suggest that trauma‐informed approaches to identifying violence exposure during prenatal care may help recognize women at increased perinatal risk, provided that privacy, trained personnel, referral pathways, and safety safeguards are in place. Because GBV may reflect ongoing household safety concerns, identification of violence during pregnancy should also prompt survivor‐centered safety planning and multidisciplinary support that considers both maternal wellbeing and infant/child safeguarding needs, in accordance with local ethical and legal frameworks. Such approaches should be implemented carefully to avoid increasing risk to the mother or child.

At the population level, the observed excess risk suggests that even modest reductions in violence exposure could translate into meaningful improvements in perinatal outcomes. Integrating violence prevention and response strategies within maternal health systems may therefore represent a promising area for intervention. However, the effectiveness of specific screening and intervention programs in reducing adverse perinatal outcomes remains insufficiently studied and warrants rigorous evaluation in future research.

### 4.4 Strengths and limitations

Key strengths of this study include its prospective design, large sample size, early pregnancy assessment of exposure, and comprehensive adjustment for relevant sociodemographic and social determinants. The use of statistical models appropriate for outcome frequency, including penalized regression for rare events, and the explicit reporting of both relative and absolute effect measures enhance the robustness and interpretability of the findings.

Several limitations should be acknowledged. First, gender‐based violence was assessed by self‐report at a single first‐trimester time point, which likely resulted in under‐ascertainment due to stigma and safety concerns. In addition, the questionnaire captured experiences occurring within the preceding 12 months but did not record exact dates; therefore, exposure could not be temporally assigned to preconception, periconceptional, or early gestational periods at the individual level. Such non‐differential exposure misclassification would be expected to bias associations toward the null. Consistent with this, sensitivity analyses exploring plausible misclassification scenarios indicated that the observed associations were robust to under‐reporting of exposure. Second, GBV was captured as a global binary screening indicator and was not disaggregated into emotional, physical, or sexual violence. This limited our ability to estimate subtype‐specific associations or determine whether particular forms of violence carried differential perinatal risks. Combining different forms of violence may have introduced exposure heterogeneity; therefore, our estimates should be interpreted as the association between any reported GBV at early pregnancy screening and perinatal outcomes, rather than as the effect of a specific violence subtype. Third, information on the persistence, frequency, timing beyond early pregnancy, and severity of violence was not available, precluding assessment of dose–response relationships or the impact of sustained exposure across pregnancy. Fourth, follow‐up of child outcomes was limited to the perinatal and neonatal periods; therefore, we could not evaluate whether prenatal GBV exposure was associated with later infant or early childhood morbidity, mortality, developmental outcomes, injury, neglect, or other safeguarding concerns. As with all observational studies, residual confounding cannot be excluded, and causal interpretations should be made cautiously. Finally, the study was conducted in a single metropolitan setting, which may limit generalizability to rural areas or regions with different sociocultural contexts.

### 4.5 Implications for future research

The present findings highlight the need for further research to clarify the mechanisms linking gender‐based violence to severe perinatal outcomes, particularly stillbirth. Future studies should aim to capture domain‐specific and longitudinal patterns of violence exposure, including emotional, physical, and sexual violence, as well as frequency, severity, and persistence across pregnancy. Expanding research to rural and underserved populations will also be essential to better characterize the full burden of gender‐based violence and its impact on perinatal health across diverse settings.

## 5 CONCLUSION

In this large urban cohort from a low‐ and middle‐income setting, gender‐based violence assessed in early pregnancy was associated with a clinically meaningful increase in adverse perinatal outcomes. This excess risk was evident on both relative and absolute scales and appeared to be driven primarily by an increased likelihood of stillbirth rather than neonatal mortality. These findings highlight gender‐based violence as an important factor to consider in the assessment of perinatal risk among pregnant women in urban LMIC contexts.

## AUTHOR CONTRIBUTIONS

Elsa Romelia Moreno‐Verduzco, Javier Pérez Duran, Irma Eloisa Monroy‐Muñoz, and Juan Mario Solis‐Paredes contributed to data collection and validation. Salvador Espino‐y‐Sosa and Raigam Martinez‐Portilla contributed to study design, statistical analysis, and critical revision of the manuscript. Johnatan Torres‐Torres conceived the study, led the analytical strategy, interpreted the results, and drafted the manuscript. All authors meet the ICMJE criteria for authorship, critically reviewed the manuscript, and approved the final version for submission.

## FUNDING INFORMATION

No external funding was received for the conduct of this study, including study design, data collection, analysis, interpretation of results, or manuscript preparation. The Instituto Nacional de Perinatología “Isidro Espinosa de los Reyes” (INPer) provided institutional support to cover the article processing charges, enabling open‐access publication. The institution had no role in the study design; data collection, analysis, or interpretation; manuscript writing; or the decision to submit the manuscript for publication.

## CONFLICT OF INTEREST STATEMENT

The authors declare no conflicts of interest.

## Supporting information


Table S1
Table S2

## Data Availability

The datasets generated and analyzed during the present study are available from the corresponding author upon reasonable request. Data access will be granted to qualified researchers for legitimate scientific use. Requests should be addressed to torresmmf@gmail.com.
