# Supplementary material for: Gender‐based violence during pregnancy and adverse perinatal outcomes in an urban low‐ and middle‐income setting: A prospective cohort study
Source: Int J Gynaecol Obstet. 2026 Jun 24;175(3):1364–73. doi: 10.1002/ijgo.71189 (PMC13769341; doi:10.1002/ijgo.71189)
Supplement: Supplementary file 1 [file IJGO-175-1364-s001.pdf]

Supplementary Table S1. STROBE Statement—Checklist of items that should be included in reports of ***cohort studies***

Gender-based violence during pregnancy and adverse perinatal outcomes in an urban low- and middle-income setting: a prospective cohort study

	Item No	Recommendation	Page No
**Title and abstract**	1	(*a*) Indicate the study's design with a commonly used term in the title or the abstract	1
		(*b*) Provide in the abstract an informative and balanced summary of what was done and what was found	2-3
**Introduction**			
Background/rationale	2	Explain the scientific background and rationale for the investigation being reported	3-4
Objectives	3	State specific objectives, including any prespecified hypotheses	4-5
**Methods**			
Study design	4	Present key elements of study design early in the paper	5-6
Setting	5	Describe the setting, locations, and relevant dates, including periods of recruitment, exposure, follow-up, and data collection	5-6
Participants	6	(*a*) Give the eligibility criteria, and the sources and methods of selection of participants. Describe methods of follow-up	5-6
		(*b*) For matched studies, give matching criteria and number of exposed and unexposed	NA
Variables	7	Clearly define all outcomes, exposures, predictors, potential confounders, and effect modifiers. Give diagnostic criteria, if applicable	6-10
Data sources/ measurement	8*	For each variable of interest, give sources of data and details of methods of assessment (measurement). Describe comparability of assessment methods if there is more than one group	6-9
Bias	9	Describe any efforts to address potential sources of bias	6-11
Study size	10	Explain how the study size was arrived at	5-6
Quantitative variables	11	Explain how quantitative variables were handled in the analyses. If applicable, describe which groupings were chosen and why	8-10
Statistical methods	12	(*a*) Describe all statistical methods, including those used to control for confounding	9-11
		(*b*) Describe any methods used to examine subgroups and interactions	NA
		(*c*) Explain how missing data were addressed	5-6
		(*d*) If applicable, explain how loss to follow-up was addressed	5-6
		(*e*) Describe any sensitivity analyses	10-11
**Results**			
Participants	13*	(a) Report numbers of individuals at each stage of study—eg numbers potentially eligible, examined for eligibility, confirmed eligible, included in the study, completing follow-up, and analysed	11
		(b) Give reasons for non-participation at each stage	NA
		(c) Consider use of a flow diagram	NA
Descriptive data	14*	(a) Give characteristics of study participants (eg demographic, clinical, social) and information on exposures and potential confounders	11-12

		(b) Indicate number of participants with missing data for each variable of interest	5-6
		(c) Summarise follow-up time (eg, average and total amount)	5-6
Outcome data	15*	Report numbers of outcome events or summary measures over time	13-14

Main results	16	(*a*) Give unadjusted estimates and, if applicable, confounder-adjusted estimates and their precision (eg, 95% confidence interval). Make clear which confounders were adjusted for and why they were included	13-14
		(*b*) Report category boundaries when continuous variables were categorized	NA
		(*c*) If relevant, consider translating estimates of relative risk into absolute risk for a meaningful time period	13
Other analyses	17	Report other analyses done—eg analyses of subgroups and interactions, and sensitivity analyses	14
**Discussion**			
Key results	18	Summarise key results with reference to study objectives	14-16
Limitations	19	Discuss limitations of the study, taking into account sources of potential bias or imprecision. Discuss both direction and magnitude of any potential bias	18-19
Interpretation	20	Give a cautious overall interpretation of results considering objectives, limitations, multiplicity of analyses, results from similar studies, and other relevant evidence	14-19
Generalisability	21	Discuss the generalisability (external validity) of the study results	19
**Other information**			
Funding	22	Give the source of funding and the role of the funders for the present study and, if applicable, for the original study on which the present article is based	20

*Give information separately for exposed and unexposed groups.

Note: An Explanation and Elaboration article discusses each checklist item and gives methodological background and published examples of transparent reporting. The STROBE checklist is best used in conjunction with this article (freely available on the Web sites of PLoS Medicine at http://www.plosmedicine.org/, Annals of Internal Medicine at http://www.annals.org/, and Epidemiology at http://www.epidem.com/). Information on the STROBE Initiative is available at http://www.strobe-statement.org.

Supplementary Table S2. Sensitivity analysis for non-differential misclassification of gender-based violence exposure

Sensitivity analysis exploring the potential impact of under-ascertainment of gender-based violence (GBV) on the association with the composite adverse perinatal outcome. Corrected estimates were derived assuming non-differential exposure misclassification under plausible ranges of Se and Sp.

Assumed sensitivity (Se)	Assumed specificity (Sp)	Corrected RR	95% CI for corrected RR	Corrected RD	Corrected RD per 1,000 births	Valid bootstrap replicates (n)
0.30	0.97	1.86	0.05 – 2.06	0.356	356	275
0.30	0.99	1.34	0.11 – 1.93	0.141	141	285
0.40	0.97	1.84	0.05 – 2.00	0.352	352	276
0.40	0.99	1.33	0.05 – 1.80	0.137	137	300
0.50	0.97	1.83	0.05 – 1.97	0.350	350	276
0.50	0.99	1.32	0.06 – 1.74	0.134	134	300
0.60	0.97	1.82	0.06 – 1.96	0.348	348	276
0.60	0.99	1.31	0.06 – 1.70	0.133	133	300

Corrected adjusted risk ratios (RRs) and risk differences (RDs) were estimated under a non-differential exposure misclassification framework using analytic correction formulas based on assumed sensitivity (Se) and specificity (Sp) of self-reported GBV exposure.

The primary analysis assumed plausible ranges of sensitivity between 0.30 and 0.60, consistent with prior literature on under-reporting of interpersonal violence in pregnancy, and high specificity (0.97–0.99), reflecting the structured nature of the questionnaire and confidential administration procedures.

Bootstrap resampling (300 repetitions) with replacement was used to assess uncertainty in corrected estimates. Replicates yielding implausible corrected risks (i.e., corrected risks <0 or >1, or undefined ratios) were excluded. The number of valid bootstrap replicates contributing to each scenario is reported in the final column.

Due to numerical instability in extreme bootstrap replicates—particularly for absolute risk differences—corrected RDs are presented as scenario-based point estimates rather than interpreted primarily through confidence intervals. Emphasis is placed on the consistency of effect direction and magnitude across plausible misclassification scenarios.

Across all evaluated scenarios, corrected adjusted RRs remained greater than unity, indicating that the association between reported GBV and adverse perinatal outcomes was robust to potential under-ascertainment of exposure.
